# Extensive complement-dependent enhancement of HIV-1 by autologous non-neutralising antibodies at early stages of infection

**DOI:** 10.1186/1742-4690-8-16

**Published:** 2011-03-14

**Authors:** Suzanne Willey, Marlén MI Aasa-Chapman, Stephen O'Farrell, Pierre Pellegrino, Ian Williams, Robin A Weiss, Stuart JD Neil

**Affiliations:** 1 MRC/UCL Centre for Medical Molecular Virology, Division of Infection and Immunity, University College London, 46 Cleveland Street, London W1T 4JF, UK; 2 Department of Infectious Diseases, King's College London, Peter Gorer Department of Immunobiology, Borough Wing, Guy's Hospital, London SE1 9RT, UK; 3 Centre for Sexual Health and HIV Research, University College London, UK

## Abstract

**Background:**

Non-neutralising antibodies to the envelope glycoprotein are elicited during acute HIV-1 infection and are abundant throughout the course of disease progression. Although these antibodies appear to have negligible effects on HIV-1 infection when assayed in standard neutralisation assays, they have the potential to exert either inhibitory or enhancing effects through interactions with complement and/or Fc receptors. Here we report that non-neutralising antibodies produced early in response to HIV-1 infection can enhance viral infectivity.

**Results:**

We investigated this complement-mediated antibody-dependent enhancement (C'-ADE) of early HIV infection by carrying out longitudinal studies with primary viruses and autologous sera derived sequentially from recently infected individuals, using a T cell line naturally expressing the complement receptor 2 (CR2; CD21). The C'-ADE was consistently observed and in some cases achieved infection-enhancing levels of greater than 350-fold, converting a low-level infection to a highly destructive one. C'-ADE activity declined as a neutralising response to the early virus emerged, but later virus isolates that had escaped the neutralising response demonstrated an increased capacity for enhanced infection by autologous antibodies. Moreover, sera with autologous enhancing activity were capable of C'ADE of heterologous viral isolates, suggesting the targeting of conserved epitopes on the envelope glycoprotein. Ectopic expression of CR2 on cell lines expressing HIV-1 receptors was sufficient to render them sensitive to C'ADE.

**Conclusions:**

Taken together, these results suggest that non-neutralising antibodies to the HIV-1 envelope that arise during acute infection are not 'passive', but in concert with complement and complement receptors may have consequences for HIV-1 dissemination and pathogenesis.

## Background

Many antibodies produced by HIV-1-infected individuals bind to the viral envelope glycoprotein, yet fail to neutralise the virus. These non-neutralising responses are usually considered 'silent' because they have little effect on HIV-1 infectivity in traditional neutralisation assays. However, antibodies also have other effector functions, including their ability to activate complement, a cascade of serum proteins that can be deposited on the virion membrane. Complement activation can lead to both viral inactivation and enhanced infection, with the latter depending on cellular expression of receptors for complement components (CRs). We have examined the effects of complement on antibodies and viruses from patients with acute HIV-1 infection using cell lines with a CR (CR2). We show that, far from being 'silent', antibodies present during acute infection can enhance viral infectivity by up to several hundred-fold, primarily by stabilising interactions between the virus and the cell. Furthermore, viruses that escape from a neutralising response remain susceptible to enhancement. Since many immune cells that HIV-1 infects or interacts with express CRs, antibody-complement interactions may play an important role in the pathogenesis of HIV/AIDS, and could be detrimental to host control of HIV-1 as well as a consideration in the evaluation of envelope-based vaccines.

## Introduction

HIV envelope-specific antibodies can be detected in the blood of infected individuals within a few weeks of infection [1,2]. In contrast, the development of a neutralising antibody response takes several months, with the timing and potency varying substantially between individuals [1,3-8]. Following the development of neutralising antibodies the virus rapidly and repeatedly escapes the induced response, so that the majority of virus is weakly, if at all, neutralised by contemporaneous antibodies [4,5,9,10]. Thus, in early stages of infection prior to the emergence of a neutralising response, non-neutralising antibodies predominate; at subsequent stages of infection, rapid escape by the virus ensures a continuing abundance of non-neutralising antibodies in the infected individual [11].

Despite the fact that non-neutralising antibodies do not directly affect viral infectivity, some of them are still able to bind to envelope proteins on the viral surface [12]. Both neutralising and non-neutralising antibodies bound to the viral surface can activate complement or bind directly to Fc receptors (FcRs) [11]. HIV can also activate complement in the absence of antibodies through direct interactions between the envelope proteins gp41 and gp120, and complement cascade components C1q and MBL [13-17], while bound antibodies amplify complement activation and the deposition of complement fragments on the viral surface [18-20]. In both the presence and absence of antibody, complement-coated virions can then interact with complement receptors (CRs) that bind C3 fragments or C1q [21]. Interactions between antibodies and FcRs, complement and CRs, and their downstream consequences, can have diverse effects on virus replication, but are largely missed in neutralisation assays due to the absence of complement in the system and lack of CRs/FcRs on target or bystander cells. In recent years, a number of antibody effector functions have been observed in early HIV infection, including antibody-dependent cell-mediated virus inhibition (ADCVI; [22,23]), and activation of the complement cascade [6,24,25]. Antibody-effector functions have been reported to both increase and compromise the efficacy of neutralising antibodies, and in the case of non-neutralising antibodies or sub-neutralising concentrations of neutralising antibodies, inhibit or enhance HIV infectivity [11].

The effect of complement, particularly, appears to be a double-edged sword. Inactivation through opsonisation and lysis have been reported [6,24,26,27], yet when CRs are present on the target cell, antibodies and complement can enhance viral infectivity [28-31]. The factors that determine the outcome of such interactions are of importance to vaccine studies as they may make the difference between a preventative and harmful vaccine candidate.

Enhanced infection of a virus opsonised with complement and antibodies via CRs on the target cell is termed complement-mediated antibody-dependent enhancement (C'-ADE). C'-ADE of HIV has been previously well-characterised [30-34], but predominantly using X4-tropic T cell line-adapted (TCLA) strains of HIV, and never, to our knowledge, using primary isolates and paired autologous antibodies from infected individuals. Enhancement of HIV by complement alone has also been reported, and this effect has been observed on primary cells and with primary strains of HIV [35-38]. Few of these studies demonstrate greater than 10-fold increases of viral infectivity, but given the long time-course of HIV infection this is considered sufficient to have a significant impact on viral dynamics. CRs so far implicated in C'-ADE of HIV include CR2 [28,39], CR3 [29] and C1qR [39], with C'-ADE via CR2 most frequently reported. Mechanistically, C'-ADE could occur by increasing physical attachments between the virus and the target cell, or through CR2-mediated signalling events leading to enhanced infection via an alternative route of entry, enhanced viral replication, or suppression of intracellular antiviral responses [40]. Current evidence favours increased attachment to the target cell [41] leading to enhanced virus entry [42].

Enhancing antibodies have been detected *in vitro *to a wide range of viruses [33,40,43], and have been linked to increased pathogenesis in dengue [44-47], Murray Valley encephalitis [48], respiratory syncytial [49], ebola [50] and measles [51] virus infections, and increased cross-placental transmission of CMV [52]. Upon viral challenge following vaccination, enhanced acquisition of infection or accelerated disease progression compared to placebo controls have been observed for the lentiviruses FIV [53-57], SIV [58,59], and EIAV [60,61]. Enhancing antibodies specific for the virus envelope proteins have been suggested, but not unequivocally proven, to play a role in vaccine-induced disease enhancement, with clearest evidence for antibody involvement coming from passive plasma transfer studies [55].

Here, we report that non-neutralising antibodies produced early in response to HIV infection can enhance viral infectivity. We investigated a role for enhancing antibodies in early HIV infection by carrying out longitudinal studies with primary viruses and autologous sera derived sequentially from recently infected individuals, using a T cell line naturally expressing CR2. We found that C'-ADE was consistent and dramatic, in some cases achieving infection-enhancing levels of greater than 350-fold. C'-ADE declined as a neutralising response to the early virus emerged, but later virus isolates that had escaped the neutralising response demonstrated an increased capacity for enhanced infection by autologous antibodies. The mechanism of enhancement was investigated by constructing cell lines expressing CR2 (CD21) or a mutant CR2 lacking a cytoplasmic tail. High-level C'-ADE occurred through both receptors, indicative of increased attachment to the target cell being the principal mechanism.

## Results

### A model system for studying C'-ADE

**Figure 1:**
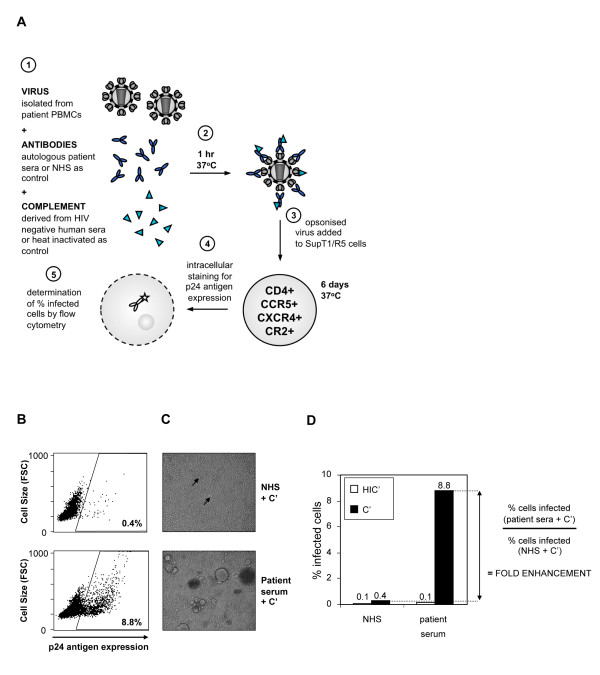
**The enhancement assay**. **(A**) Schematic diagram of the assay. **(B) **Flow cytometry dot plots from a typical enhancement assay. The gate is set against uninfected control SupT1/R5 cells. The upper panel shows the percentage of SupT1/R5 cells infected by virus pre-incubated with C' and NHS control serum, the lower panel with C' and an example autologous patient serum. **(C) **Light microscopy images from a typical enhancement assay. Images were taken of the cells analysed in **1B**, prior to intracellular p24 staining and flow cytometry. The upper panel shows cells infected by virus pre-incubated with C' and NHS control serum, the lower panel with C' and autologous patient serum. Arrows on the upper panel indicate the presence of syncytia, which are large and numerous on the lower panel. **(D) **Calculation of fold enhancement. All experiments are performed alongside an HIV-antibody-negative (NHS) control culture, in the presence (C') and absence (HIC') of active complement. Enhancement is observed in the presence of C', therefore fold enhancement is calculated as the ratio of infected cells in the presence of C' and test (patient) serum to infected cells in the presence of C' and NHS. Figures 1B, C and D are all derived from experiments using MM32.10 virus and MM32 day 15 autologous serum (see Figure 2). The NHS + C' control experiments yielded 0.4% infected cells and the patient serum + C' cultures 8.8% infected cells, equating to a 22-fold enhancement of infection.

Previous C'-ADE studies have been restricted by the target cell used. Commonly used T cell lines, such as MT-2, do not support infection by clinically relevant R5 tropic primary isolates, whereas assays performed on primary cells have the inherent problem of long preparation methods and donor variability. Furthermore, complement control proteins are at least partially responsible for observed evasion of complement-mediated lysis of HIV virions and are expressed on primary CD4+ T cells [62-64]. Therefore, we used PBMC-derived virus isolates in order to produce virus that closely resembled that produced *in vivo*, and developed a novel assay system to study C'-ADE of primary isolates, using the T cell line SupT1/R5, which naturally expresses CD4, CXCR4 and CR2, and has been transduced to stably express CCR5 (Figure 1A). Viruses were incubated with antibody (heat-inactivated patient serum) and complement (pooled fresh seronegative human serum; C'), both at a final concentration of 10%, for 1 hour at 37°C and then added to the SupT1/R5 cells (Figure 1A). Control experiments were performed in parallel in which the patient sera were replaced with pooled seronegative normal human serum (NHS; antibody-negative control). In addition to this, the C' was replaced with a heat-inactivated equivalent (HIC'; complement-negative control) in assays of both patient sera and NHS in order to detect the antibody-only mediated effects on viral replication (e.g. neutralisation). Infection was detected by intracellular p24 staining and flow cytometry 6 days after inoculation, and the percentage of infected cells calculated.

Flow cytometry plots, microscopy images and fold enhancement calculations from a typical enhancement assay are shown in Figure 1B, C and 1D. In all experiments, results are reported as *fold enhancement*, meaning the ratio of infection in the presence of autologous (patient) serum to infection in the presence of NHS, calculated separately for infection in the presence of HIC' and C' (Figure 1D). This cancels out the complement-only enhancement in the assays, which was a virus-specific effect that ranged from 2 to 10-fold for the viruses used in this study (Table 1; also evident in Figure 1D), and allows the data to be presented only in terms of the additional effect of the HIV-specific antibodies. All serum samples were assayed at a final concentration of 10% in order to represent the dominant antibody activity in the sera, rather than diluting sera (as is common practice in some enhancement assays), which may skew results by under-representing neutralisation. Only fold increases that exceeded the stringent cut-off of 5-fold were scored as enhanced infection, as normal human serum taken from nine uninfected individuals gave a mean fold enhancement of 1.85 (range 0.72 - 3.7; standard deviation from the mean 0.9) compared to the pooled control serum, NHS.

**Table 1:** Patient primary virus isolates

Patient	Virus	Day of virus isolation	C'-only enhancement (fold)
MM24	MM24.26	26	2.2
	MM24.464	464	2

MM25	MM25.18	18	2.4

MM26	MM26.62	62	2.1
	MM26.384	384	3.6

MM27	MM27.28	28	2
	MM27.585	585	3.8

MM28	MM28.6	6	2.1

MM32	MM32.10	10	3.1

MM33	MM33.12	12	3.4

MM34	MM34.32	32	9.7
	MM34.443	443	2.1

MM38	MM38.29	29	3.8

MM42	MM42.29	29	1.9
	MM42.238	238	2

Virus isolates are named according to patient ID (e.g. MM24) followed by a decimal point then the day of virus isolation. Days represent the time following onset of symptoms indicative of acute HIV infection. Viruses were isolated from patient PBMCs by co-culture, and expanded by minimal passage in fresh PBMCs. Fold increases in infection seen in the presence of complement alone (C'; i.e. in the absence of HIV-specific antibodies) compared to in the presence of heat-inactivated complement (HIC') are shown for each virus.

### High-level enhancement of early patient isolates by early autologous sera

The primary focus of this study was to investigate the occurrence of C'-ADE in early HIV infection in the time-frame between seroconversion and the development of an autologous neutralising response - a time of dynamic virus replication and antibody production, during which non-neutralising antibodies dominate in the infected individual [65]. Viruses were isolated from 10 HIV-1 clade B-infected individuals from the Jenner cohort [1,6] at the earliest time points available, between 6 and 62 days following onset of symptoms characteristic of primary HIV infection (DFOS; Table 1), and assayed in the presence of sequential autologous serum samples and exogenous human C' or HIC'.

**Figure 2:**
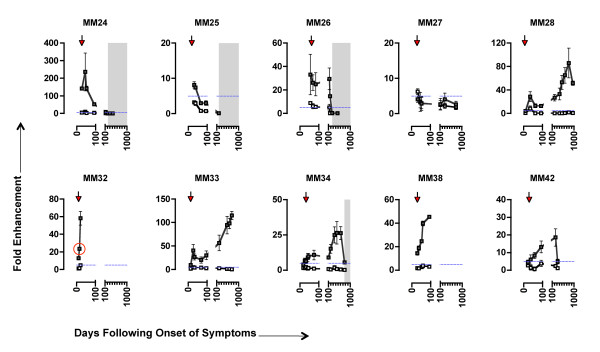
**High-level C'-ADE of early viruses by autologous sequential sera**. Serum sets from 10 patients were tested against autologous virus for C'-ADE activity. Sera and virus time points are shown as days following onset of symptoms characteristic of primary HIV infection (DFOS). Arrows indicate the time point from which virus was isolated. White squares represent assays conducted in the presence of HIC'; grey squares, C'. Error bars represent standard deviations from 3 experiments. To show clearly events pre-day 100 (during which the antibody response is developing rapidly and sampling is more frequent), breaks have been inserted into the X-axes. Post day-100 intervals = 100 days. Shading indicates the first appearance and continued detection of an autologous neutralising antibody response (as determined by >90% neutralisation in an independent neutralisation assay). The horizontal dashed blue line indicates the cut-off point for positive C'-ADE. Note that the scales of the Y-axes differ between subjects. The datum point equating to the enhancement depicted in **Figure 1 B**, **C **and **D **(MM32.10 virus with day 15 autologous serum) is circled in red.

Anti-envelope antibodies were detectable (by ELISA) in all individuals and increased steadily over time (Additional File 1**; Figures S1A and S1B**). Total IgG and IgM were also measured and were within or higher than the expected range for healthy individuals (data not shown). The C'-ADE assay design was optimised for measuring increases in infection, therefore independent neutralisation assays were carried out on paired plasma samples from each individual (in the absence of C') in order to characterise the development of a neutralising response in detail (Table 2). The results from the longitudinal C'-ADE assays and a summary of the neutralisation data are shown in Figure 2. With the exception of MM27, all patients showed evidence of high-level C'-ADE activity (grey squares), with enhancement levels reaching 236-fold (MM24.26 virus with day 44 serum). The enhancing effect of the patient sera was complement-dependent, as the same sera assayed in the presence of HIC' had only minimal effect on replication (white squares). The detection of a neutralising response to the early virus, as defined by >90% inhibition compared to control cultures in the independent neutralisation assays (shaded areas), coincided with the complete disappearance (MM24, MM25, MM26) or a sharp drop (MM34) of C'-ADE activity.

**Table 2:** Neutralisation of early patient viruses by sequential autologous plasma

Patient	MM24								
**DFOS**	**44**	**93**	**124**	**208**	**292**	**409**	**464**	**555**	**834**
**IC90**	< 10	< 10	< 10	80	320	640	640	640	640
**IC50**	10	10	10	320	1280	1280	1280	2560	1280

**Patient**	**MM25**								

**DFOS**	**31**	**38**	**66**	**94**	**185**				
**IC90**	< 10	< 10	< 10	< 10	10				
**IC50**	< 10	< 10	< 10	< 10	40				

**Patient**	**MM26**								

**DFOS**	**55**	**76**	**83**	**111**	**139**	**169**	**253**	**337**	**474**
**IC90**	< 10	< 10	< 10	< 10	< 10	< 10	10	40	80
**IC50**	< 10	< 10	< 10	< 10	< 10	10	40	160	320

**Patient**	**MM27**								

**DFOS**	**46**	**53**	**109**	**299**	**585**	**755**			
**IC90**	< 10	< 10	< 10	< 10	< 10	< 10			
**IC50**	< 10	< 10	< 10	< 10	< 10	< 10			

**Patient**	**MM28**								

**DFOS**	**20**	**34**	**62**	**93**	**198**	**405**	**503**	**782**	**950**
**IC90**	< 10	< 10	< 10	< 10	< 10	< 10	< 10	< 10	< 10
**IC50**	< 10	< 10	< 10	< 10	< 10	< 10	< 10	< 10	20

**Patient**	**MM32**								

**DFOS**	**10**	**14**	**21**						
**IC90**	< 10	< 10	< 10						
**IC50**	< 10	< 10	< 10						

**Patient**	**MM33**								

**DFOS**	**26**	**33**	**69**	**96**	**201**	**523**	**621**	**719**	
**IC90**	< 10	< 10	< 10	< 10	< 10	< 10	< 10	< 10	
**IC50**	< 10	< 10	< 10	< 10	< 10	< 10	< 10	< 10	

**Patient**	**MM34**								

**DFOS**	**32**	**45**	**74**	**192**	**443**	**607**	**759**		
**IC90**	< 10	< 10	< 10	< 10	< 10	< 10	10		
**IC50**	< 10	< 10	< 10	< 10	< 10	< 10	10		

**Patient**	**MM38**								

**DFOS**	**29**	**36**	**51**	**58**	**93**				
**IC90**	< 10	< 10	< 10	< 10	< 10				
**IC50**	< 10	< 10	< 10	< 10	< 10				

**Patient**	**MM42**								

**DFOS**	**22**	**29**	**36**	**43**	**57**	**92**	**183**	**238**	**324**
**IC90**	< 10	< 10	< 10	< 10	< 10	< 10	< 10	< 10	< 10
**IC50**	< 10	< 10	< 10	< 10	< 10	< 10	< 10	< 10	< 10

Early patient viruses (detailed in **Table 1**) and sequential autologous plasma samples were analysed by standard TZMbl neutralisation assay. Plasma time points are represented by DFOS (days following onset of symptoms) as for serum samples used throughout this study. IC50 and IC90 values represent the highest plasma dilution at which reductions in infection of 50 and 90%, respectively, are achieved relative to control cultures.

We discerned three patterns of C'-ADE of early viruses. MM24, MM25 and MM26 showed strongest C'-ADE at early time points, with C'-ADE subsiding sharply upon the appearance of a neutralising response. Neutralisation occurred between 185 and 253 days following onset of symptoms (Figure 2 and Table 2). The magnitude of the C'-ADE differed between the three individuals, with MM24 showing the highest peak levels (236-fold on day 44), and MM25 the lowest (8-fold on day 31). In contrast to this, MM28, MM33, MM34 and MM42 displayed C'-ADE for an extended period of time, with peak C'-ADE occurring between days 200 and 800, and increases of C'-ADE over time paralleling the increased production of anti-Env antibodies (Figure 2 and Additional File 1**; Figure S1**). In these individuals, there was also a minor peak of C'-ADE activity before day 50, analogous to the early peak seen for MM24, MM25 and MM26. For MM34, the emergence of a neutralising response coincided with a reduction in C'-ADE activity on day 759 (Figure 2 and Table 2). Neutralising activity was not detected for MM28, MM33 and MM42 in the time-frame investigated (Table 2). As with the first group of individuals (MM24, MM25 and MM26), the magnitude of the C'-ADE differed between these individuals, with MM33 showing the highest peak levels (115-fold on day 719) and MM42 the lowest (19-fold on day 238). C'-ADE was also detected with early virus and sera from MM32 and MM38, but their longitudinal patterns of C'-ADE could not be determined due to study opt-out and commencement of antiretroviral therapy, respectively. In contrast to all other patients, C'-ADE of MM27 was variable and below the 5-fold threshold for genuine C'-ADE (Figure 2).

The observed C'-ADE activity emerges at the same time as antibodies previously shown to have C'-mediated inactivation (C'-MI) activity [6]. However, as lysis was not considered the principal mechanism of C'-MI it is possible that the same antibodies can mediate both C'-ADE and C'-MI effects, but that the outcome is determined by the target cell. In order to place the observed C'-ADE activity in the context of the various antibody functions observed at early time points following infection, Additional File 2**; Figure S2 **shows the longitudinal C'-ADE, C'-MI and neutralisation profiles for one of the patients (MM26). Both C'-ADE and C'-MI are seen prior to the development of neutralising antibodies, hence non-neutralising antibodies produced early in infection can mediate both C'MI and C'ADE, although it cannot be excluded that the distinct activities of the whole sera tested in different assay conditions may be attributable to different types of antibodies within the sera.

To relate our results back to previously reported C'-ADE studies, we tested a known enhancing monoclonal antibody (mAb), 246-D, in our system. 246-D enhanced infection of the TCLA strain IIIB up to 3.7-fold, comparable to previous reports with enhancing mAbs [34,39], and showed only limited enhancement of the patient isolate MM38.29 (Additional File 3**; Figure S3**). As a control, a known neutralising mAb, IgGb12, was tested alongside 246-D and expected neutralisation results were obtained.

For further comparisons with previous C'-ADE studies, serum samples from MM24, MM25, MM26 and MM27 were tested with IIIB. Fold enhancement levels of no greater than 6-fold were observed (data not shown), also consistent with previous reports of C'-ADE [25,28,41,66], and demonstrating that the high level of enhancement seen with the patient isolates is not a universal feature of our system.

### C'-ADE increases the number of cells infected, virus output and cell death

The enhanced infection evident in Figure 1 and 2 was also detectable when infection was measured by alternative methods, including reverse transcriptase (RT) output as a measure of virus production and percentage (%) cell loss (Table 3 and Additional File 4**; Figure S4**). C'-ADE transformed a low-level infection (0.2% cells infected; 0% cell loss compared with uninfected control cultures) to a highly destructive one (46% cells infected; 80% cell loss compared with uninfected control cultures). The increased detection of virus (RT) in the cell supernatant over time (Additional File 4**; Figure S4A**), along with clear evidence of cytopathic effect (Figure 1C), indicates increased productive infection and ongoing virus replication in the enhanced cultures.

**Table 3:** Enhancement is characterised by increased cell infection, increased virus production and increased cell death

Parameter	Assay	NHS + C'	Day 44 serum + C'	Fold enhancement
% infected cells	Intracellular p24 stain	0.20	46.1	236
% viable cells	MTT	101	20	N/A
RT production (pg/ml)	RT ELISA	222	20495	92

To further characterise the enhancement and elucidate whether the flow cytometry results obtained were representative of productive infection, reverse transcriptase (RT) output (to quantitate virus production) and percentage (%) cell death (normalised to uninfected control cultures) were measured by RT ELISA and MTT assay, respectively. Results shown are from enhanced and control infection of MM24.26 virus. Experiments were carried out in the presence of C', with fold enhancement calculated by dividing infection levels in the presence of patient serum (MM24 day 44) by infection in the presence of control serum (NHS).

### The observed C'-ADE was mediated by CR2, with increased attachment to the target cell the primary mechanism

Limited investigation has been carried out on the mechanisms of C'-ADE occurring through CR2, and the ability of CR2 to mediate these effects on various cell types. Potential ways in which C'-ADE could occur through CR2 include: increased attachment of the virus to the target cell, resulting in increased efficiency of entry; signalling through the receptor resulting in endocytosis of the virus and subsequent infection via an alternative pathway; signalling through the receptor to suppress intracellular antiviral activity; or signalling through the receptor to increase viral replication.

**Figure 3:**
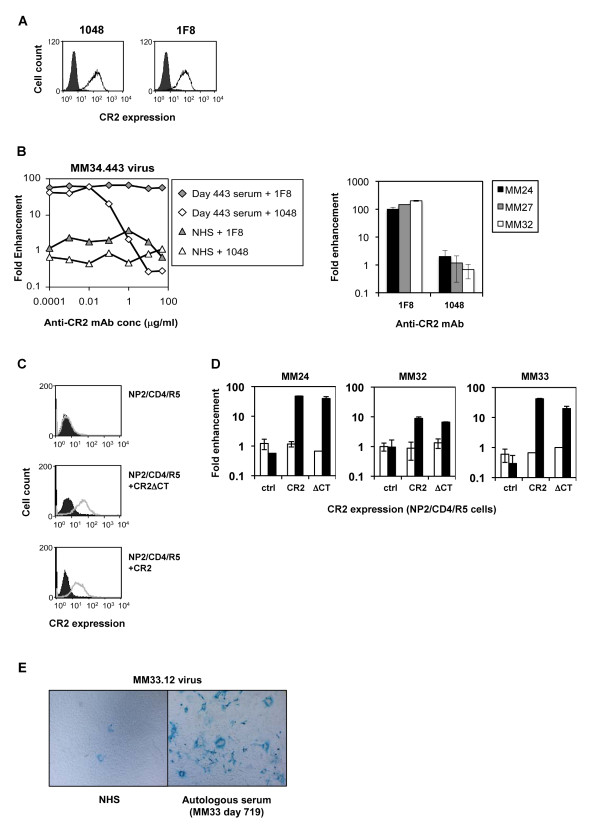
**C'-ADE is mediated by CR2 and can also be mediated by a mutant CR2 lacking a cytoplasmic tail**. **(A) **Binding of two anti-CR2 mAbs, 1048 and 1F8, to SupT1/R5 cells was tested by flow cytometry. Both antibodies recognise a C3d-binding region-containing portion of CR2, but only 1048 blocks ligand binding. Both are shown to bind SupT1/R5 cells, at 10 μg/ml. **(B) **Left panel: the involvement of CR2 in C'-ADE was initially investigated by pre-incubating SupT1/R5 cells with increasing concentrations of C3dg ligand-blocking mAb 1048, and non-blocking mAb 1F8 as a control, before performing enhancement assays as usual, with MM34.443 virus and day 443 autologous serum. Right panel: a further three subjects (MM24.464 virus with day 292 serum; MM27.585 virus with day 299 serum; MM32.10 virus with day 21 serum) were tested in the presence of 10 μg/ml 1F8 or 1048 mAb. All experiments were performed in the presence of C', and fold enhancement was calculated relative to infection in the presence of NHS + C' and in the absence of anti-CR2 mAbs. **(C) **NP2/CD4/R5 cells stably expressing CR2 and CR2ΔCT were established by retroviral vector transduction. Expression levels were determined by flow cytometry using an anti-CR2 mAb. **(D) **Enhancement experiments were carried out on NP2/CD4/R5 (control; ctrl), NP2/CD4/R5/CR2 (CR2) and NP2/CD4/R5/CR2Δcytoplasmic tail (ΔCT) cells using MM24.464 virus opsonised with day 292 autologous serum, MM32.10 virus opsonised with day 15 autologous serum, and MM33.12 virus opsonised with day 719 autologous serum, and HIC' (white bars) or C' (black bars). Fold enhancement was calculated relative to infection in the presence of NHS + HIC' or NHS + C', as appropriate, on each cell line. Error bars represent standard deviations from 3 experiments. **(E) **Images of the enhanced infection of MM33.12 virus by day 719 autologous serum on NP2/CD4/R5/CR2 cells. The left-hand image shows infection in the presence of NHS + C', and the right-hand image infection in the presence of day 719 serum + C'.

In order to formally demonstrate that the C'-ADE observed was mediated by CR2, and to exclude the involvement of other molecules on the SupT1/R5 cells, we performed studies using the mAb 1048, known to block the binding of the complement factor C3 d to CR2 [67], and the anti-CR2 mAb 1F8 as a control, which binds to CR2 but does not prevent its C3 d binding activity [68]. Both antibodies recognised CR2 on the SupT1/R5 cells (Figure 3A). Cells were incubated with increasing concentrations of 1048 or 1F8 for 30 minutes at room temperature, washed, then used as target cells in enhancement assays. Initial anti-CR2 mAb titration experiments using MM34.443 virus and day 443 autologous serum demonstrated that 1048 completely abolished C'-ADE activity at concentrations greater than 1 μg/ml, whereas 1F8 had no effect on infection or C'-ADE up to 50 μg/ml (Figure 3B). A further three subjects (MM24.464 virus with MM24 day 292 serum; MM27.585 virus with MM27 day 299 serum; and MM32.10 virus with MM32 day 21 serum) were then tested at C'-ADE-inhibitory concentrations of the blocking mAb (10 μg/ml). As before, for all three subjects the 1F8 control mAb had no effect on C'-ADE, while the blocking mAb 1048 abrogated C'-ADE activity (Figure 3B).

To investigate the role of receptor signalling, and thus the mechanism of C'-ADE, full-length and truncated CR2 (lacking the cytoplasmic tail, ΔCT) constructs were cloned and expressed in the HIV-permissive cell line NP2/CD4/R5 (Figure 3C). One late patient virus, MM24.464, and two early patient viruses, MM32.10 and MM33.12, were opsonised with known enhancing autologous sera, MM24 day 292, MM32 day 15 and MM33 day 719 respectively, plus HIC' or C', before addition to NP2/CD4/R5 control, CR2+ or ΔCT+ cells. High-level C'-ADE of all three viruses occurred on the CR2+ and ΔCT+ cells; C'-ADE did not occur on the control cells (Figure 3D). Infection with MM24.464 was enhanced 48-fold on the CR2+ cells and 40-fold on the ΔCT+ cells, compared with 189-fold in the equivalent SupT1/R5 assay. Infection with MM32.10 virus was enhanced 9-fold on the CR2+ cells and 7-fold on the ΔCT+ cells, compared with 24-fold in the equivalent SupT1/R5 assay (Figure 2). Infection with MM33.12 was enhanced 42-fold on the CR2+ cells (light microscopy images of which are shown in Figure 3E) and 20-fold on the ΔCT+ cells; equivalent enhancement assays carried out on SupT1/R5 cells resulted in a 115-fold enhancement (Figure 2). As C'-ADE can occur in the absence of the CR2 cytoplasmic tail, signalling processes through CR2 are not an essential part of the C'-ADE mechanism. Enhanced infection, through increased attachment of the virus to the target cell, is, therefore, likely to be the principal mechanism of C'-ADE.

### C'-ADE activity is present in both IgG and IgM plasma fractions, but IgG showed the most potent C'-ADE activity

**Figure 4:**
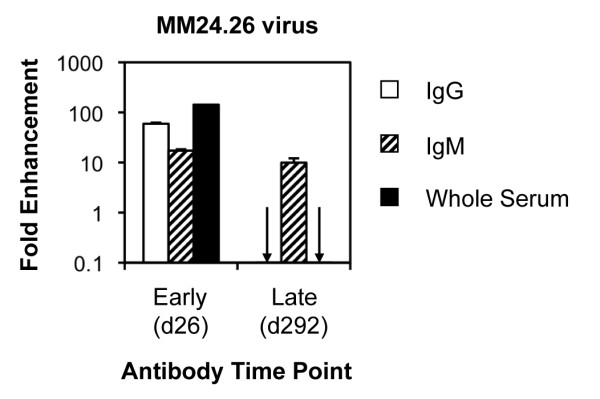
**C'-ADE activity of purified IgG and IgM**. IgG and IgM were purified from early (day 26) and late (day 292) plasma from MM24 and tested against MM24.26 virus in the presence of C' on SupT1/R5 cells. Fold enhancement was calculated relative to infection in the presence of IgG or IgM purified from NHP, or whole NHS, as appropriate, and C'. Error bars represent standard deviations from 3 experiments.

To confirm that the C'-ADE mediated by patient serum was attributable to the immunoglobulin fraction, and to investigate the contributions of the IgG and IgM fractions, IgG and IgM were purified from early, late and seronegative control plasma (NHP) in parallel. Both IgG and IgM purified from early (day 26) plasma from MM24 were capable of enhancing infection of the autologous early virus (MM24.26), with the IgG enhancing to a greater magnitude (Figure 4). Late (day 292) IgM continued to enhance MM24.26, whereas the late IgG, in keeping with the late serum, neutralised MM24.26.

### Later virus isolates from the same individuals are enhanced to a greater degree than early isolates

**Figure 5:**
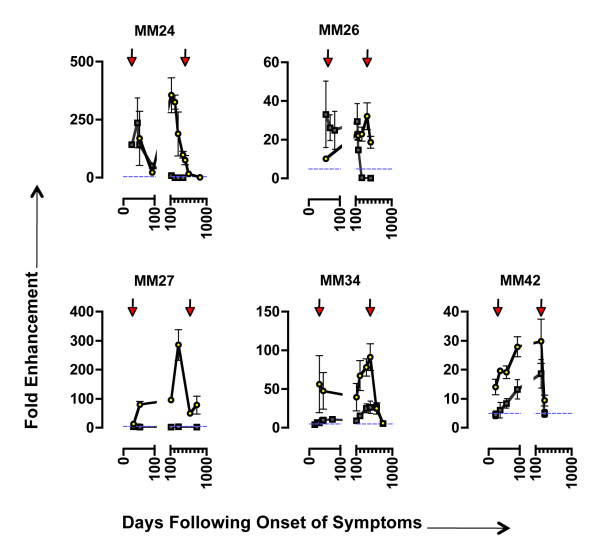
**C'-ADE of early and late viruses from 5 patients by autologous sequential sera**. Viruses isolated at early and late time points following onset of symptoms were assayed on SupT1/R5 cells in the presence of C' and sequential autologous sera. Arrows indicate early and late virus isolation times. Grey squares represent assays performed with early virus (as in Figure 2); yellow circles with late virus. The horizontal dashed blue line indicates the cut-off point for positive C'-ADE. Note that the scales of the Y-axes differ between subjects. Error bars represent standard deviations from 3 experiments.

In the face of an emerging neutralising antibody response, virus evolution in infected individuals is rapid and ongoing [4,5,9,10]. We therefore investigated whether virus isolates from chronic infection (taken from between 238 and 585 days following onset of symptoms; Table 1) from 5 individuals maintained the capacity for enhanced infection. Two later viruses were tested from individuals that showed strong early C'-ADE and then neutralisation of early virus (MM24 and MM26; Figure 2), one from the individual that did not show C'-ADE of early virus (MM27; Figure 2), and two from individuals that showed C'-ADE of early virus for an extended period of time with peak levels post day 200 (MM34 and MM42; Figure 2). C'-ADE profiles for both early and late viruses from these five individuals are shown in Figure 5.

With the exception of MM24.464 virus, for all of the later viruses tested the peak C'-ADE activity occurred in the serum sample obtained on or immediately before the day the virus was isolated. For MM24.464, the peak C'-ADE activity occurred 340 days earlier with day 124 serum (356-fold enhancement). For both MM24 and MM26, sera that neutralised the early viruses enhanced the later viruses. C'-ADE activity of MM24.464 virus was 326-fold with day 208 serum, whereas day 208 serum neutralised MM24.26 virus. Similarly, the peak C'-ADE activity of MM26.384 virus was 32-fold with day 384 serum, whereas day 384 serum potently neutralised MM26.62 virus. Although no C'-ADE was detected when the early MM27 virus (MM27.28) was assayed, the later MM27 virus (MM27.585) was enhanced by autologous sera, peaking at 286-fold with day 299 serum. Thus, although MM27 serum does not enhance MM27.28 virus, it does not lack C'-ADE activity. For MM34 and MM42, the same profile of C'-ADE seen for the early viruses was maintained for the later viruses, but at an overall higher magnitude. C'-ADE peaked on day 443 for MM34, with MM34.32 enhanced 27-fold and MM34.443 enhanced 92-fold. For MM42 C'-ADE peaked on day 238, with MM42.28 enhanced 19-fold and MM42.238 enhanced 30-fold.

With the exception of patient MM24, whose early virus MM24.26 was already enhanced to a high level by contemporaneous serum (143-fold), later virus isolates from infected individuals were enhanced by contemporaneous sera to a significantly greater degree than early isolates (Table 4). Even later viruses that did not appear to escape a neutralising response (as NAbs were not detected by the time of isolation) were enhanced to a greater extent. Importantly, for MM34 and MM42 the patterns of C'-ADE over time were the same, but for the later viruses the magnitude of fold enhancement was greater. These findings indicate that the reason for the increased enhancement is not necessarily that the later viruses are less susceptible to neutralisation (i.e. that levels of enhancement are inversely related to levels of neutralising antibodies in sera), but that other properties of the later viruses make them more susceptible to enhancement.

**Table 4:** Comparison of C'-ADE levels in contemporaneous autologous virus-serum pairs for early and late viruses

Patient	Day of virus isolation	Contemporaneous serum enhancement	*p *value
**MM24**	26	143	N/A*
	464	76	

**MM26**	62	18	0.02
	384	32	

**MM27**	28	4	0.008
	585	50	

**MM34**	32	7	< 0.001
	443	92	

**MM42**	29	6	< 0.001
	238	30	

* decreased, rather than increased, C'-ADE

Fold enhancement (in the presence of C') is shown for contemporaneous serum with early and late viruses. P values are derived from a two-tailed Student's T test assuming equal variance, based on the null hypothesis that the two means are equal. All but one of the later virus isolates (MM24.464) are enhanced to a level significantly greater than that of the early viruses when tested with paired contemporaneous serum.

### Further characterisation of enhancing and neutralising IgG

**Figure 6:**
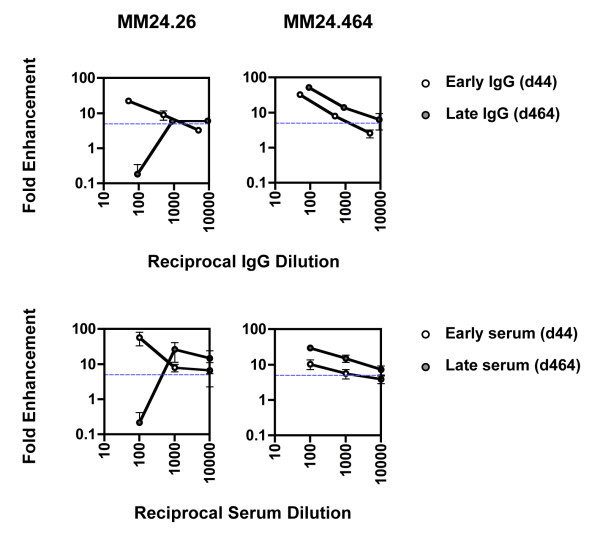
**Titration of antibodies purified from enhancing and neutralising serum**. IgG was purified by Protein G affinity chromatography from patient plasma taken on the same day as sera that enhanced and neutralised early patient virus, days 44 and 464 respectively. 10-fold serial dilutions of d44 and d464 IgG were tested against early autologous virus (MM24.26) and late autologous virus (MM24.464). The X-axes indicate IgG dilutions assayed relative to the IgG concentration in the original plasma source. Fold enhancement was calculated relative to infection in the presence of IgG purified from NHP. Identical assays performed with sera in the place of purified IgG are shown in the bottom panel for comparison. The horizontal dashed blue line indicates the cut-off point for positive C'-ADE. Error bars represent standard deviations from 3 experiments.

As shown previously in Figure 4 IgG purified from MM24 early and late plasma enhanced and neutralised MM24.26 virus, respectively. To further characterise the neutralising and enhancing activity in early and late plasma against both early and late viruses, IgG was purified from MM24 day 44 and 464 plasma (different time points from those in Figure 4 were used due to limited availability of material) and used in titration experiments. IgG concentrations in the plasma samples used for the purification, and the IgG eluted from the purification columns, were determined by IgG ELISA and the dilutions of purified IgG used in the assays adjusted relative to the original plasma IgG concentration. As expected, at the highest concentration tested, early and late MM24 IgG enhanced and neutralised MM24.26 virus, respectively, reflecting the properties of the patient sera (Figure 2 MM24 and Figure 6 MM24.26). Upon dilution, the early, enhancing IgG became less enhancing, whereas the late, neutralising IgG gained enhancing activity (Figure 6 MM24.26). Both early and late IgG enhanced the later MM24.464 virus, and this enhancing activity declined with dilution (Figure 6 MM24.464). These data, along with the temporal disappearance of C'-ADE upon the detection of a neutralising response (Figure 2), indicate that the presence of a robust neutralising activity can mask underlying enhancing activity.

### Heterologous cross-reactivity of C'-ADE but not neutralisation

**Figure 7:**
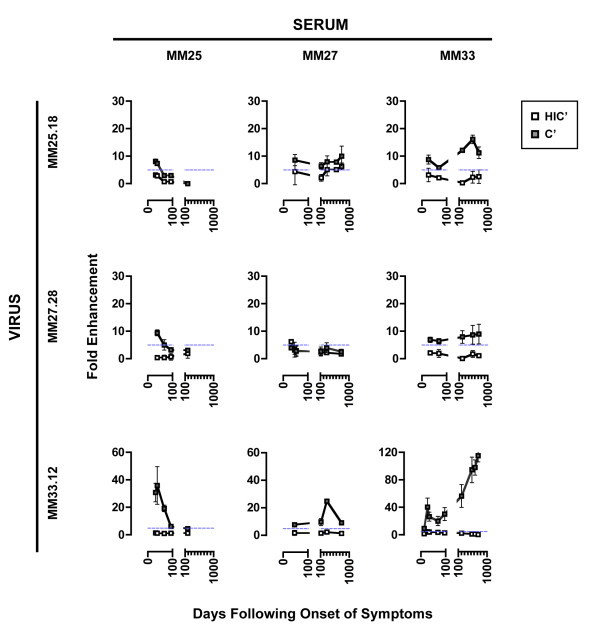
**C'-ADE by heterologous sera**. Early viruses and sequential serum sets from 3 patients were tested against each other to investigate heterologous C'-ADE. The patient from whom the virus was derived is indicated on the far left, and serum at the top. White squares represent assays conducted in the presence of HIC'; grey squares, C'. The horizontal dashed blue line indicates the cut-off point for positive C'-ADE. Note that the scales of the Y-axes differ between subjects. Error bars represent standard deviations from 3 experiments.

To investigate the breadth of the C'-ADE response, virus-sera sets from three patients (MM25, MM27 and MM33; Figure 2) were assessed for heterologous C'-ADE activity (Figure 7). The heterologous C'-ADE profiles show that the pattern of C'-ADE over time was a property of the serum whilst the magnitude of the C'-ADE was a property of the virus. The MM27.28 virus was interesting as this virus was not enhanced by autologous sera, yet was enhanced by MM25 and MM33 sera, albeit at the low levels of 9- and 10- fold respectively. Conversely, MM27 sera could enhance other viruses, with peaks of 25-fold for MM33.12 virus and 10-fold for MM25.18. This implies that characteristics of both MM27 virus and sera together, rather than either alone, limited C'-ADE in the autologous assays. While all sera tested that showed C'-ADE of autologous virus also showed C'-ADE of heterologous virus, the MM25 day 185 serum that neutralised autologous virus did not show cross-neutralising activity of heterologous viruses.

## Discussion

Here we have shown that infectivity of primary isolates from individuals with early HIV infection can be enhanced over 350-fold when opsonised with autologous antibodies and complement, compared with infection in the presence of complement alone. The range of increases in infection are comparable to those reported for dengue virus, a disease in which ADE contributes to pathogenesis during secondary infection by a different serotype and in infants carrying sub-neutralising levels of maternal antibodies [44,46,47,69,70]. Previous studies using X4-tropic TCLA strains of HIV, or primary isolates opsonised with complement alone, typically showed up to 10-fold increases in infection [25,28,36,41,42,66], as we have also confirmed when performing similar experiments. A well-characterised enhancing mAb, 246-D, enhanced infection of the TCLA HIV strain IIIB by up to 3.7-fold in our assay system, whereas the same mAb showed a very modest enhancement of the patient primary isolate MM38.29. The same patient primary isolate was enhanced up to 46-fold by autologous serum. We attribute the high level of C'-ADE shown in this study to the use of primary clinical isolates of HIV and their autologous antibodies on T cells able to support infection by R5-tropic viruses. The high levels reported here suggest that C'-ADE may be an important infection-enhancing mechanism *in vivo*. Furthermore, access to a cohort of recently infected individuals allowed us to perform longitudinal studies of C'-ADE and provided us with a unique perspective of the development of this response over time. Later virus isolates were enhanced more than early isolates from the same individuals, indicating that the C'-ADE observed in our model system reflects a replicative advantage for the enhanced viruses *in vivo*.

Most HIV antibody assays *in vitro *are carried out in the absence of complement, yet, *in vivo*, cell-free HIV is likely to be opsonised with antibodies and complement at all stages of disease post-seroconversion [71]. Given that complement can modulate antibody activity, it is logical to consider the effects of complement alongside any study of antibodies. Furthermore, HIV replicates predominantly in lymphoid tissues, which are rich in immune cells expressing CRs, including macrophages, DCs, and some T cells and B cells [11,71]. We have shown that the presence of a CR on an HIV target cell can drastically alter the outcome of virus opsonisation with antibodies and complement. We have shown that sera with apparently no activity when assayed on CR-negative cells (Table 2 and Figure 3), or with virus-inhibitory activity when assayed on CR-negative cells in the presence of complement (Additional File 2**; Figure S2**), can enhance infection by several orders of magnitude on CR-positive cells in the presence of complement (both on T cells naturally expressing CR2, and on unrelated cells engineered to express CR2; Figure 2, Figure 3, Additional File 2**; Figure S2**).

The C'-ADE observed in our model system could represent a variety of processes mediated through CRs *in vivo*. Through analogy with previous reports carried out with virus opsonised with complement alone, these could include direct infection-enhancement of CR+ target cells, enhanced B cell-to-T cell or DC-to-T cell *trans*-infection, or enhanced FDC trapping of complement- and antibody-opsonised virions [35-38,72-77], all of which could influence HIV pathogenesis. For example, CR2 has been implicated in HIV trapping, archiving and *trans*-infection of T cells mediated by B cells and FDCs [73-78]. The high levels of enhancement seen here through CR2 may reflect an important role for non-neutralising, enhancing antibodies in these processes *in vivo*, and may be particularly relevant to early B cell-mediated viral dissemination and subsequent FDC trapping. In addition to B cells and FDCs, CR2 expression has been reported on T cell subsets [79-84], thymocytes [82,85,86] and astrocytes [87], which may support directly enhanced infection by HIV.

The principal complement component ligands of CR2 are the C3 fragments C3dg and C3 d, and to a lesser extent iC3b [71,88]. The enhancement reported here through CR2 might also occur through other receptors for C3 complement fragments, namely CR1, CR3 and CR4 [71]. Complement alone has been shown to substantially increase infection of primary monocytes/macrophages and DCs expressing CRs [35-38], and enhancing antibodies may augment this process by increasing the deposition of complement on the virion. However, effects mediated through CRs may be counteracted by reported virus-inhibitory effects mediated through FcRs on macrophages and DCs [37,89], and the net outcome may be influenced by the receptor balance on the target cell and the nature of the antibodies [36,37]. Future evaluation of CR and FcR expression on primary cell targets for HIV, and investigation of the role of these receptors in enhancing and inhibiting HIV infection, is warranted.

In agreement with results obtained by Montefiori and colleagues in a macaque model of acute SIV infection [90], the presence or emergence of neutralising activity masks pre-existing enhancing activity. This is shown here by the temporal disappearance of enhancing activity upon the detection of a potent neutralising response (Figure 2** and **Table 2), and by detection of enhancement upon dilution of neutralising IgG (Figure 6). Mechanistically, this could be because abundant non-neutralising antibodies enhance infection through increasing attachment to the target cell, while the neutralising antibodies are ultimately able to block entry of the virus if present in sufficient quantities, whether it is through enhanced or direct infection.

Our studies of heterologous virus-serum pairs show that enhancing, but not neutralising, activity can be transferred to heterologous viruses, perhaps because the enhancing antibodies are directed to more conserved, non-neutralising, immunodominant epitopes. Yet, this does not equate with any antibody having the potential to enhance - it is likely that antibodies must be of a reasonable affinity and concentration for enhancement to occur. Early studies of C'-ADE of HIV demonstrated an association between gp41 antibodies and C'-ADE activity [91,92]. A key "enhancing domain" was identified within the PID of gp41, in a region primarily accessible on disassembled or post-fusion envelope spikes [93]. The association between C'-ADE and gp41 antibodies may be of particular relevance to the acute stage of HIV infection, when the first antibodies produced in response to infection are directed against gp41 and are non-neutralising [2].

Laboratory preparations of HIV, and cell-free HIV present in plasma of infected individuals, are often attributed with low infectivity relative to the number of physical virus particles present, with discrepancies between infectious units and physical virus particles estimated to be as high as 1 to 60,000 [94-96]. The traditional explanation for this is a high level of "defective" viral particles, produced as a consequence of the error-prone nature of reverse transcriptase and the labile nature of the viral envelope proteins. An alternative explanation is that, due to a relatively low efficiency of initial cellular receptor engagement [97], coupled with the low number of trimeric envelope spikes on the viral surface [98], the infectivity of HIV is underestimated because the vast majority of virus particles in a preparation lack the opportunity to infect the target cells [99]. This is supported by other demonstrations of dramatic enhancement of HIV infectivity *in vitro*, through coating of HIV with semen amyloid fibrils [100], incorporation of host attachment proteins into the viral membrane [101], and direct interactions between gp120 and C-type lectin receptors on target or bystander cells [102,103]; all are mechanisms that stabilise the virus on the target cell and improve the efficiency of receptor interaction. It is unclear to what extent these mechanisms would function *in vivo *when the virus is coated with antibodies and complement. The possibility of C'-ADE also drawing on the so-called "non-infectious" pool of virions, as opposed to increasing the replication of readily infectious virions, is supported by our studies of the mechanism of C'-ADE, which show that a version of CR2 lacking a cytoplasmic tail (and consequently lacking signalling ability) supports high-level C'-ADE. Under these conditions, increased attachment to the target cell is the most likely mechanism of C'-ADE.

In conclusion, our study highlights the possibility that antibodies with seemingly no activity in standard cell-based neutralisation assays may have other activities when assayed under conditions that accommodate effector functions, and may even contribute to viral pathogenesis.

## Materials and methods

### Study subjects

Blood samples were obtained from a cohort of individuals recently infected with HIV-1 subtype B, as previously described [1,6]. All individuals were men who have sex with men (MSM) presenting with primary HIV illness (PHI) following sexual exposure to HIV. Blood samples were obtained weekly for the first month, monthly for 3 months and subsequently every 3 months. Plasma, serum, and whole blood were divided into aliquots and stored at -80°C. PBMCs were isolated by Ficoll gradient centrifugation and stored in liquid nitrogen. The study protocol was approved by the Camden and Islington NHS Trust Ethics Committee and written informed consent obtained from all subjects. Approval for the use of human serum, plasma and peripheral blood leukocytes from healthy volunteers was granted to Professor R. Weiss by the University College London Committee on Ethics (project ID 0335/001).

### Patient sera, control sera, complement and monoclonal antibodies

The patient serum samples used as an antibody source were aliquoted and heat inactivated (56°C for 1 hour) before use, and complement (referred to as C' in the assays) was added back into the system as an exogenous source in the form of pooled serum from four HIV-seronegative individuals. Due to the labile nature of complement proteins, pooled complement was aliquoted and immediately stored at -80°C for a maximum of 3 months, and thawed only once before use. In all assays containing complement, a heat-inactivated equivalent (HIC') was used in parallel as a control. The HIV antibody-negative control serum (normal human serum; NHS), was pooled commercial human serum (PAA Laboratories, Somerset, UK), also heat inactivated for 1 hour at 56°C, aliquoted and stored at -80°C until use. Previously characterised positive control sera (QC sera [104]), used in ELISAs, were from three individuals with chronic HIV infection, and were also heat inactivated and aliquoted before use. The anti-gp120 mAb IgGb12 [105] was kindly provided by D. Burton, Scripps Institute, La Jolla, US. mAb 4E10 [106] was obtained from Polymun Scientific GmbH, Vienna, Austria. 246-D [91] was obtained through the NIH AIDS Research and Reference Reagent Program, NIH, US, from S. Zolla-Pazner.

### Viruses

All patient primary isolates were propagated in PBMCs with minimal passage. PBMCs were isolated by Ficoll gradient centrifugation from fresh blood drawn from healthy HIV-negative volunteers. Purified PBMCs were stimulated with 0.5 μg/ml PHA and cultured for 2 days, then treated with 5 U/ml human IL-2 for a further 3 days. 1-2 × 10^7 ^cells from two different donors were used for co-culture with 5 × 10^6 ^unstimulated patient PBMCs (collected from whole blood of infected individuals by Ficoll gradient centrifugation at the time points specified) or inoculation with 1 ml virus supernatant, for 2 hours at 37°C. Fresh growth medium was added and the cells incubated at 37°C for 7 days. On days 8, 15 and 22 post-inoculation, cells were split, cell supernatants were cleared by centrifugation, and cultures were replenished with fresh PBMCs, prepared as above. Viral production was monitored using a commercial p24 ELISA (Biomerieux, France), and virus titres from peak days of p24 production were determined on NP2/CD4/CCR5 and NP2/CD4/CXCR4 cells, as detailed below. The TCLA virus IIIB was propagated in the T cell line H9.

### Cell lines

The human glioma cells lines NP2/CD4/CCR5 and NP2/CD4/CXCR4, described elsewhere [107], were kindly provided by H. Hoshino. SupT1 cells transduced to stably express CCR5 were kindly provided by J. Hoxie. TZMbl, H9, C8166 and Molt-4 cells were obtained from NIBSC, UK. MT-2 and MT-4 cells were obtained from D. Richman through the AIDS Research and Reference Reagent Program, Division of AIDS, NIAID, NIH.

### Detection of anti-gp120 antibodies

Antibodies to gp120 in patient sera were detected by ELISA as previously described [1]. Briefly, 96-well Maxisorb plates (Nalgene, Nunc International) were coated with 10 μg/ml sheep polyclonal anti-gp120 antibody (D7324; Aalto Bio Reagents, Dublin, Ireland) overnight, blocked with 1% milk powder, and saturated with HIV-1_IIIB _gp120 (EVA657, NIBSC, UK). For each serum sample to be tested, a blank well excluding the gp120 was included. Sera were serially tenfold diluted in TMT/GS (Tris-buffered saline containing 0.05% Tween 20, 4% milk powder and 10% goat serum), added to the plate and incubated for 1 hour at room temperature. Bound antibodies were detected with alkaline phosphatase (AP)-conjugated goat anti-human Ig (Harlan SeraLab, Crawley Down, UK), followed by AP substrate solution (LumiPhos Plus, Aureon Biosystems, Vienna, Austria). Relative light units were determined at 405 nm. QC sera (pooled sera from 3 chronically infected individuals, [104]), and the anti-gp120 mAb IgGb12, were used as positive controls to enable the standardisation of readings from different plates for direct comparison of antibody levels between patients.

### Detection of anti-gp41 antibodiess

96-well Maxisorb plates (Nalgene, Nunc International) were coated with 0.05 μg/well recombinant gp41 (derived from IIIB; ARP680 NIBSC) in 0.1 M sodium bicarbonate coating buffer, washed in TBS-T (0.05% Tween in 1× TBS), blocked with TMT/GS (10% goat serum and 2% milk powder in TBS-T), washed in TBS-T and incubated with patient or control serum diluted 1 in 1,000, 5,000 and 25,000 in TMT/GS, for 1 hour at room temperature. For each serum sample to be tested, a blank well excluding the gp41 was included for background subtraction. As for the gp120 ELISA, pooled QC serum [104]) and an anti-gp41 mAb, 4E10, were used as positive controls and to standardise readings from different plates.

### Titration of virus stocks

NP2/CD4/R5 and NP2/CD4/X4 cells were seeded in 48-well plates one day prior to infection, at a density of 2 × 10^4 ^cells/well. Tenfold serial dilutions of viral stocks were added to the cells in triplicate and incubated for 2 hours at 37°C. Cells were then washed, overlaid with 500 μl culture medium and incubated for 48 hours at 37°C. Culture medium was then removed and the cells fixed for 10 min at room temperature in methanol/acetone (1:1 v/v), pre-cooled at -40°C, followed by washing in PBS. *In situ *p24 staining was used to detect infection, as described previously [1]. Viral titres, expressed as focus forming units (FFU)/ml, were determined by counting foci by light microscopy.

### Neutralisation and C'-MI assays

Neutralisation assays were carried out using two different methods: our NP2/CD4/R5-based assay, as previously described [1,6], and a widely adopted TZMbl-based assay [108]. For the TZMbl assays, a viral input of 200 TCID_50 _and incubation time of 48 hours were used. % neutralisation was calculated relative to the virus control, with the IC90 values reported being the last dilution of each plasma sample to give greater than 90% reduction of infection.

For the NP2/CD4/R5 assay, 200 focus-forming units (FFU) of virus were incubated with patient plasma or normal human plasma (NHP) as a control, in a final volume of 100 μl for 1 hour at 37°C. Patient plasma and NHP were each used at a standard concentration of 10% at the time of incubation. Medium was removed from cells and replaced with the virus-plasma mixtures, and incubated for 2 hours at 37°C. Cells were then washed once in culture medium, replenished with fresh culture medium and incubated for 48 hours at 37°C. Cells were then fixed and stained *in situ *for p24 expression [1] and foci were counted by light microscopy. % neutralisation was calculated relative to the NHP control.

Complement-mediated inactivation (C'-MI) assays were performed in a similar way to the NP2/CD4/R5 neutralisation assays [6], with the exception that patient serum (rather than patient plasma) was used as an antibody source, and NHS used as a control. Complement (C'), or heat-inactivated complement as a control (HIC'), was also added at a final concentration of 10% (v/v) [6]. % inactivation was calculated relative to the NHS control.

### SupT1/R5 enhancement assay

Virus (input equivalent to an endpoint of 0.1% infected cells in the presence of NHS and HIC') was incubated with C' (or HIC' as a complement-negative control) and patient serum (or NHS as an antibody-negative control) in a total volume of 100 μl for 1 hour at 37°C. When patient sera were serially diluted, or when mAbs were used as an antibody source, dilutions were performed in NHS to maintain a consistent level of serum in the assay. C'/HIC' and patient serum/NHS, as appropriate, were each used at a final concentration of 10% at the time of incubation. Virus-serum mixtures were next transferred to SupT1/R5 cells, seeded at 10^5 ^cells per well in U-bottomed 96-well tissue culture plates, giving a final volume of 200 μl per well. 100 μl of cell supernatant was removed on days 1 and 3 after inoculation for RT ELISA analysis (Cavidi, Sweden), and replaced with 100 μl fresh culture medium. On day 6 after inoculation, all cell supernatant was removed and stored for RT ELISA analysis, and the cells were stained for intracellular p24 expression and subsequent FACS analysis.

### Intracellular p24 stain, determination of percent infected cells and calculation of fold enhancement

Cells were fixed in 3.8% formaldehyde solution, washed once in staining buffer (PBS with 1% FCS and 0.1% sodium azide) and once in permeabilisation buffer (PERM buffer; staining buffer with 0.5% (w/v) saponin, Sigma-Aldrich). Blocking was carried out with PERM buffer containing 10% goat serum for 30 minutes. Primary antibody (an equal mixture of two mouse mAbs to two separate epitopes on p24; ADP 365 and 366, AIDS Reagent Program, NIBSC, UK) was added at a final dilution of 1:40 and incubated for 1 hour at 4°C. Cells were then washed three times in PERM buffer then incubated for 30 min at 4°C with secondary antibody (goat anti-mouse polyclonal antibody conjugated to fluorescein isothiocyanate (FITC), Dako, Denmark). Following a further two washes with PERM buffer and one wash with staining buffer, the cells were fixed again with formaldehyde solution and analysed on a FACSCalibur flow cytometer (Beckton Dickinson, UK) using Cellquest software (Beckton Dickinson, UK). Uninfected, stained cells were used as controls to set up gates for infected cells. Samples were "blanked" by averaging the number of events occurring in the infected gate for 6 uninfected samples (usually averaging <0.01%) and removing this from each test sample. Fold enhancement was determined by dividing % infected cells in the presence of test (patient) serum by % infected cells in the presence of control (NHS) serum. This was calculated separately for experiments performed in the presence (C') and absence (HIC') of active complement. True enhancement was set at the level of 5-fold, as serum taken from 7 uninfected individuals enhanced infection up to 3.7 fold ± 0.57 (compared to pooled control serum, NHS).

### CR2 antibody-blocking assays

To assess the involvement of CR2 in C'-ADE on SupT1/R5 cells, anti-CR2 mAbs were bound to SupT1/R5 cells prior to standard enhancement assays. The mouse anti-human CD21 mAb 1048 (BD Biosciences, Belgium) recognises the short consensus repeat (SCR) 1-2 region of CR2 and has previously been shown to block C3dg binding to CR2 and displace HIV-1-containing immune complexes from B cells [67]. The mouse anti-human CD21 antibody 1F8 (Dako, Denmark), also recognising a C3d-binding fragment of CR2, but does not inhibit C3 d binding [68]. 1048 and 1F8 were diluted in cell culture medium and incubated with 10^5 ^SupT1/R5 cells at room temperature at final concentrations of 50, 10, 1, 0.1, 0.01, 0.001 and 0.0001 μg/ml. Cells were then washed twice and used for enhancement assays as usual. As the anti-CR2 mAbs bound to the cellular surface might interfere with the intracellular p24 staining procedure, fold enhancement was determined by RT ELISA of the cell supernatant on day 6 following inoculation.

### RT ELISA

RT activity in cell-free supernatant was determined using the Lenti-RT Activity Assay (Cavidi Tech, Sweden), according to the manufacturer's instructions. Absorbance at 405 nm was measured in a Lucy 1 luminometer (Anthos-Labtech, UK) and analysed using Stingray software (Dazdaq, East Sussex, UK).

### IgG and IgM purification

IgG and IgM were purified from patient plasma using MabTrap protein G Columns and HiTrap IgM Purification Kit (Amersham Biosciences, UK), respectively, according to the manufacturer's instructions. IgG and IgM were purified from HIV seronegative plasma (NHP) in parallel for use as controls in the enhancement assay. When patient IgG were serially diluted, dilutions were performed in IgG purified from NHP (after first ensuring that IgG from NHP gave a similar level of infection to assays performed with NHS) to maintain a consistent level of antibodies in the assay. Elution fractions were tested for immunoglobulin content by BCA protein assay (Thermo Fisher Scientific, UK), pooled, and reconstituted to the original volume of plasma used for the purification in order to normalise results between samples. Antibody concentrations in both the purified antibody preparation and the original plasma source were determined by commercial ELISA (Zeptometrix Corporation, US) according to the manufacturer's instructions. The concentration of the purified antibody was between 5- and 9-fold less than the concentration of antibody in the plasma. Purified antibodies were diluted relative to physiological concentrations of the original plasma, or used at stated concentrations.

### Construction of CR2-expressing NP2/CD4/CCR5 cells

CR2 (CD21; gene accession number NM_001877) was amplified from SupT1 cell cDNA (prepared from cellular mRNA using an Invitrogen Superscript III kit) by polymerase chain reaction (PCR) with primers 5'- GCGCTGATCAGCCACCATGGGCGCCGCGGGCC-3' and 5'-GCGCTTCGAATCAGCTGGCTGGGTTGTAT-3'. A mutant form of CR2 lacking all but 3 amino acids (KHR) of the cytoplasmic tail was constructed by inserting a stop codon after the KHR sequence [109], using an alternative reverse primer 5'-GCGCTTCGAATCATCTGTGTTTTGATATCACGTAT-3'. PCR was performed using the AccuPrime *Taq *DNA Polymerase High Fidelity kit (Invitrogen, UK). PCR products were ligated into the cloning vector pCR2.1 TOPO (Invitrogen, UK). The CR2 and CR2ΔCT constructs were then subcloned into the lentiviral reporter construct pCSGW [110,111], with the eGFP-encoding gene removed, as *Bam*HI*/Not *I fragments (N.B. partial restriction digests were necessary due to an internal *Bam*HI site in the CR2 coding sequence). The CR2- or CR2ΔCT-encoding plasmids were co-transfected into subconfluent 293T cells with the packaging and envelope plasmids pCMVΔ8.2 and pVSV-G to produce lentiviral vectors, as described elsewhere [111]. NP2/CD4/CCR5 cells stably expressing CR2 or CR2ΔCT were produced by lentiviral vector transduction, and expression of the full-length and mutant receptors on the cell surface was detected by flow cytometry.

## Competing interests

The authors declare that they have no competing interests.

## Authors' contributions

SW designed and performed the experiments, analysed the data and wrote the manuscript. SJDN designed the experiments, interpreted results and contributed to writing the manuscript. MMIAC isolated some of the viruses, interpreted results and contributed to designing the experiments and writing the manuscript. RAW conceived the study, interpreted results and critically read the manuscript. IW, PP and SoF managed the clinical aspect of the study.

## Supplementary Material

Additional file 1 **Supplementary Figure 1. Patient antibody profiles**. **(A) **Anti-gp120 antibody levels were determined by a gp120 ELISA based on gp120 from IIIB. Antibody levels were standardised to pooled positive control serum from chronically-infected individuals, indicated on the graphs by the dashed horizontal line, to allow direct comparisons between individuals. Results shown are from sera diluted 1:100, except pooled positive control sera, assayed at 1:1000. All seronegative samples tested gave negative relative light unit (RLU) readings following background subtraction. The monoclonal anti-gp120 antibody IgGb12, when used in the same ELISA at 125 ng/ml, gave an RLU output of 1016. **(B) **Anti-gp41 antibody levels were determined by a gp41 ELISA based on the IIIB gp41. Antibody levels were standardised to pooled serum from chronically-infected individuals, indicated on the graphs by the upper dashed horizontal line. Results shown are from sera diluted 1:25,000. Lower dashed horizontal lines indicate seronegative samples. The monoclonal anti-gp41 antibody 4E10 when used in the same ELISA at 0.4 μg/ml gave an RLU output of 770. Click here for file

Additional file 2 **Supplementary Figure 2. The co-existence of C'-ADE and C'-MI activity in early serum samples from an infected individual**. C'-MI, C'-ADE and neutralisation assays were carried out using early virus (MM26.62) and sequential autologous serum samples from MM26. Anti-gp120 antibody levels were measured in sequential serum samples from MM26 by ELISA. **(A) **Anti-gp120 antibody levels in MM26 serum, detected by binding to HIV-1 IIIB gp120 in ELISA and expressed as RLU. **(B) **Neutralisation assay on NP2/CD4/R5 cells. Percentage neutralisation is calculated relative to infection in the presence of NHS. **(C) **C'-MI assay on NP2/CD4/R5 cells. Percentage inactivation is calculated relative to infection in the presence of NHS and C'. **(D) **Enhancement assay on SupT1/R5 cells. Fold enhancement is calculated relative to infection in the presence of NHS and C'. Note that all assays were carried out with the same sera and virus whilst the target cell and presence of C' differs between them. Click here for file

Additional file 3 **Supplementary Figure 3. C'-ADE by monoclonal antibodies**. The enhancing mAb 246-D and the neutralising mAb IgGb12 were serially diluted in NHS then incubated with C' or HIC' and virus, as per the standard enhancement assays. Fold enhancement is calculated relative to infection in the presence of NHS and C', as for other enhancement assays. Results are shown for IIIB (top panels) and patient primary isolate MM38.29 (bottom panels). White squares represent assays conducted in the presence of HIC'; black squares, C'. Click here for file

Additional file 4 **Supplementary Figure 4. RT correlation and enhancement assay time course**. In addition to % cells infected, fold enhancement was also measured using RT detected in the cell supernatant as a surrogate marker of virus production. **(A) **RT output was monitored in the cellular supernatant on days 1, 3 and 6 of enhancement assays for viruses showing high-level (MM34.32) and low-level (MM25.18) enhancement. Results are shown for MM25.18 virus with day 31 serum or NHS (left) and MM34.32 virus with day 25 serum or NHS (right). MM25.18 virus: when measured by RT output, day 31 serum enhanced infection 5.1-fold in the presence of C' (black triangles; 639 pg/ml RT) compared to NHS + C' (black squares; 127 pg/ml; 8.16-fold when measured by % cells infected, see Figure 2); whereas complement alone (NHS + C'; black squares; 127 pg/ml RT) enhanced infection 1-fold compared to NHS + HIC' (white squares; 125 pg/ml RT; 2.4-fold when measured by % cells infected, see Table 1). MM34.32 virus: when measured by RT output, day 25 serum enhanced infection 10-fold in the presence of C' (black triangles; 11,798 pg/ml RT) compared to NHS + C' (black squares; 1,183 pg/ml RT; 9-fold when measured by % cells infected, see Figure 2); whereas complement alone (NHS + C'; black squares; 1,183 pg/ml RT) enhanced infection 7.3-fold compared to NHS + HIC' (white squares; 162 pg/ml RT; 9.7-fold when measured by % cells infected, see Table 1). **(B) **Fold enhancement calculated from % cells infected by MM24.26 in the presence of 8 autologous sequential serum samples and complement (see Figure 2, MM24) was compared with fold enhancement attained by the same serum samples but calculated from RT output. Click here for file
