# Supplementary figures and images for: Extensive complement-dependent enhancement of HIV-1 by autologous non-neutralising antibodies at early stages of infection
Source: Retrovirology. 2011 Mar 14;8:16. doi: 10.1186/1742-4690-8-16 (PMC3065417; doi:10.1186/1742-4690-8-16)

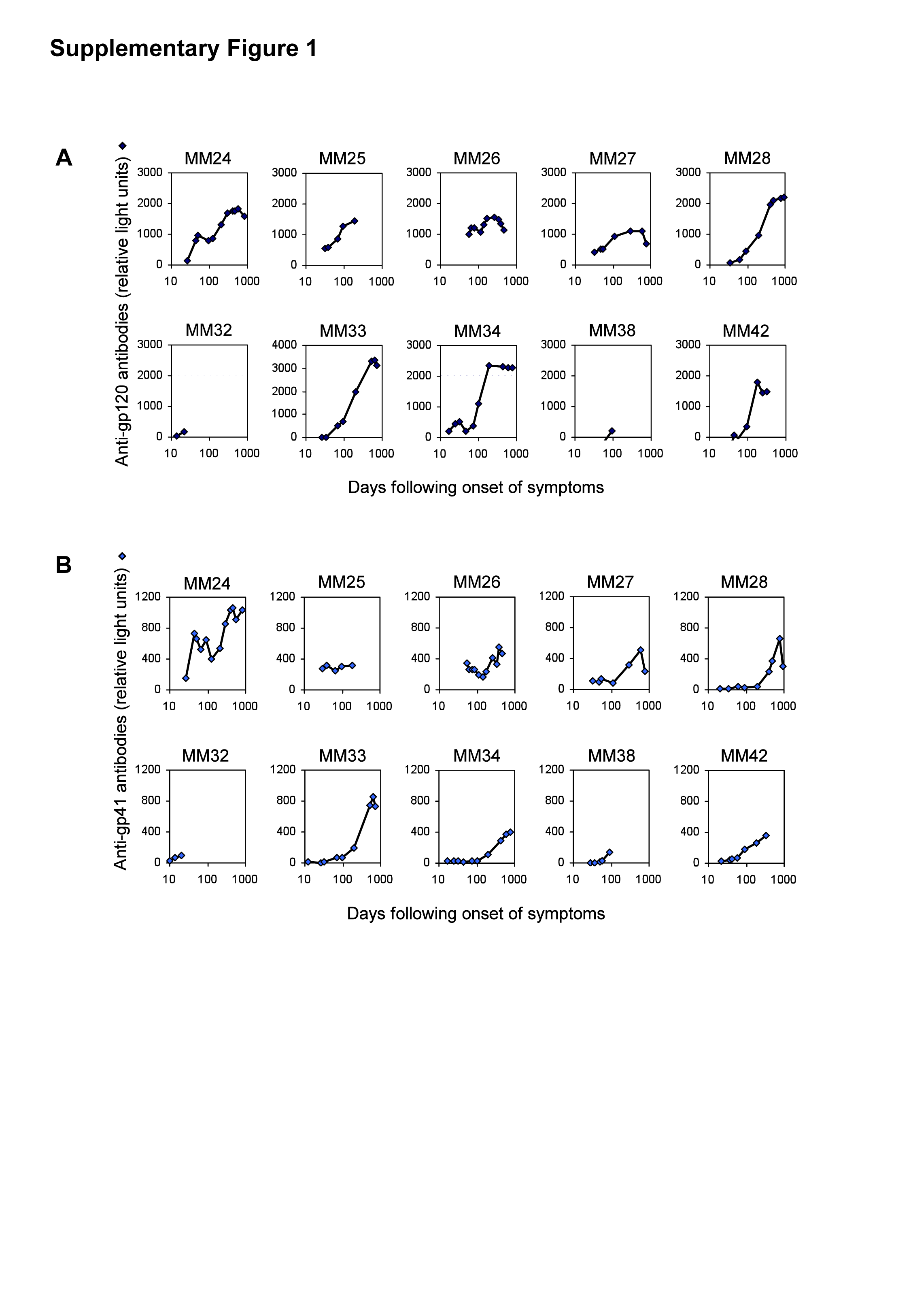

Supplement: Additional file 1 — Supplementary Figure 1. Patient antibody profiles. (A) Anti-gp120 antibody levels were determined by a gp120 ELISA based on gp120 from IIIB. Antibody levels were standardised to pooled positive control serum from chronically-infected individuals, indicated on the graphs by the dashed horizontal line, to allow direct comparisons between individuals. Results shown are from sera diluted 1:100, except pooled positive control sera, assayed at 1:1000. All seronegative samples tested gave negative relative light unit (RLU) readings following background subtraction. The monoclonal anti-gp120 antibody IgGb12, when used in the same ELISA at 125 ng/ml, gave an RLU output of 1016. (B) Anti-gp41 antibody levels were determined by a gp41 ELISA based on the IIIB gp41. Antibody levels were standardised to pooled serum from chronically-infected individuals, indicated on the graphs by the upper dashed horizontal line. Results shown are from sera diluted 1:25,000. Lower dashed horizontal lines indicate seronegative samples. The monoclonal anti-gp41 antibody 4E10 when used in the same ELISA at 0.4 μg/ml gave an RLU output of 770. [file 1742-4690-8-16-S1.TIFF]

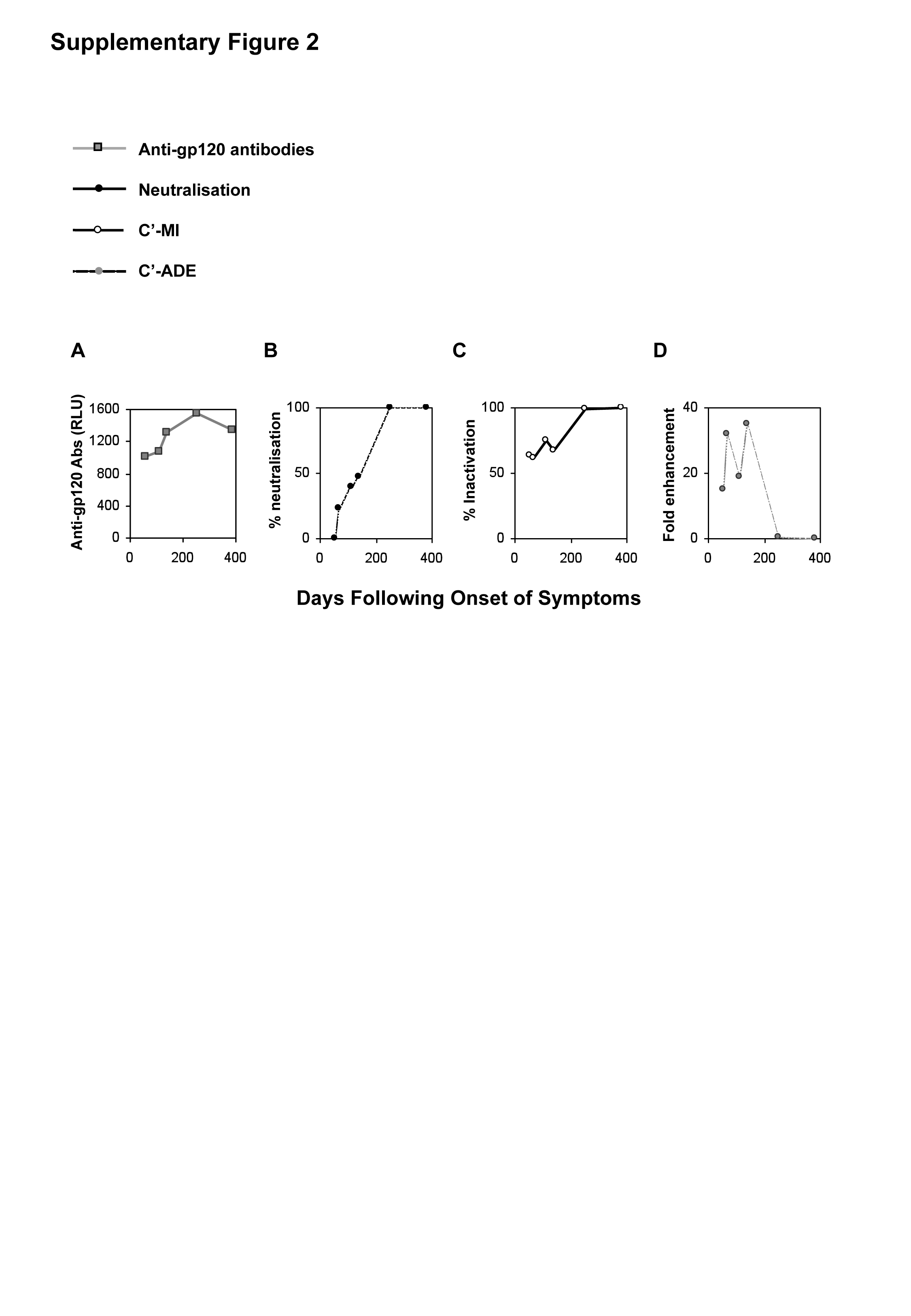

Supplement: Additional file 2 — Supplementary Figure 2. The co-existence of C'-ADE and C'-MI activity in early serum samples from an infected individual. C'-MI, C'-ADE and neutralisation assays were carried out using early virus (MM26.62) and sequential autologous serum samples from MM26. Anti-gp120 antibody levels were measured in sequential serum samples from MM26 by ELISA. (A) Anti-gp120 antibody levels in MM26 serum, detected by binding to HIV-1 IIIB gp120 in ELISA and expressed as RLU. (B) Neutralisation assay on NP2/CD4/R5 cells. Percentage neutralisation is calculated relative to infection in the presence of NHS. (C) C'-MI assay on NP2/CD4/R5 cells. Percentage inactivation is calculated relative to infection in the presence of NHS and C'. (D) Enhancement assay on SupT1/R5 cells. Fold enhancement is calculated relative to infection in the presence of NHS and C'. Note that all assays were carried out with the same sera and virus whilst the target cell and presence of C' differs between them. [file 1742-4690-8-16-S2.TIFF]

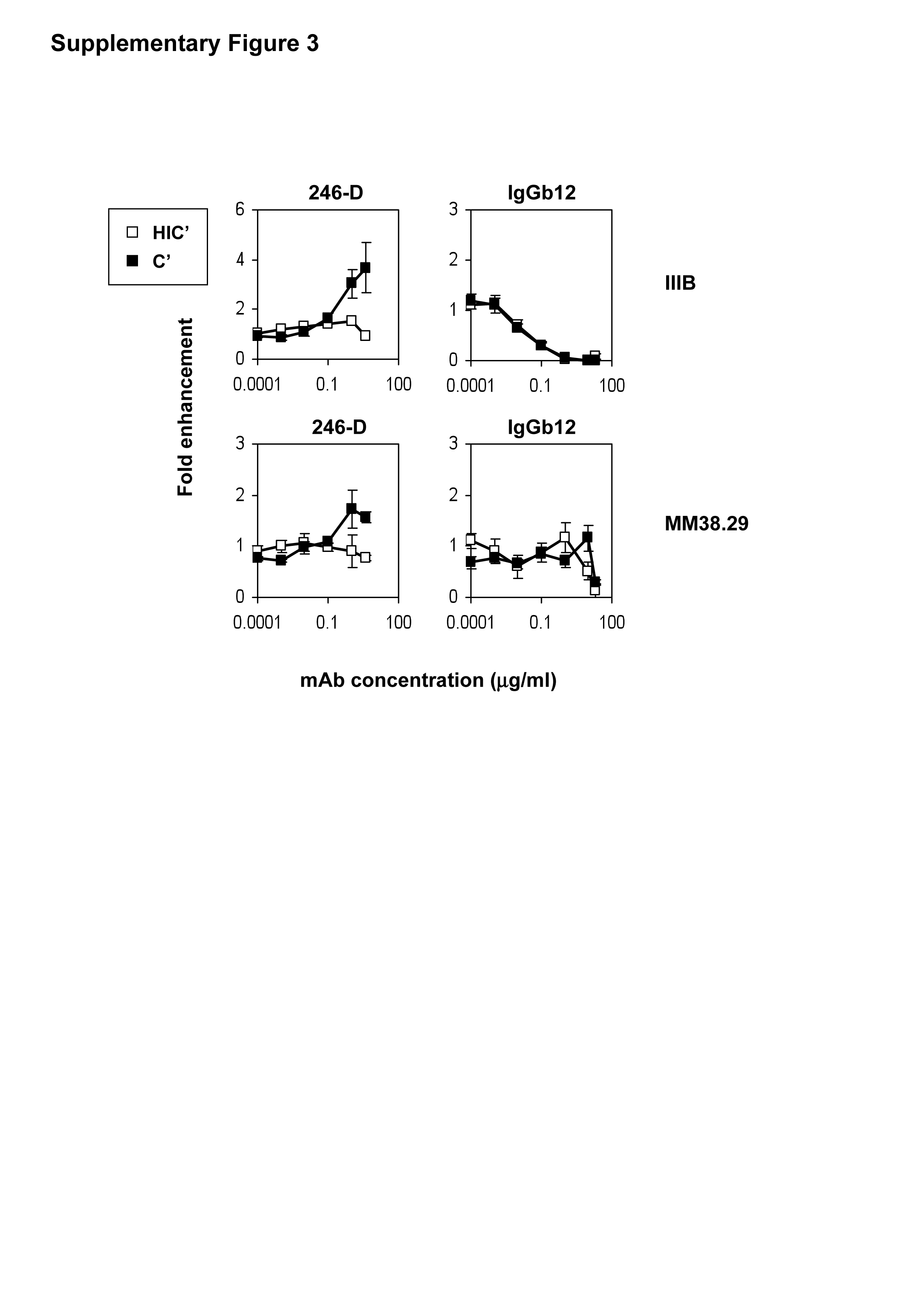

Supplement: Additional file 3 — Supplementary Figure 3. C'-ADE by monoclonal antibodies. The enhancing mAb 246-D and the neutralising mAb IgGb12 were serially diluted in NHS then incubated with C' or HIC' and virus, as per the standard enhancement assays. Fold enhancement is calculated relative to infection in the presence of NHS and C', as for other enhancement assays. Results are shown for IIIB (top panels) and patient primary isolate MM38.29 (bottom panels). White squares represent assays conducted in the presence of HIC'; black squares, C'. [file 1742-4690-8-16-S3.TIFF]

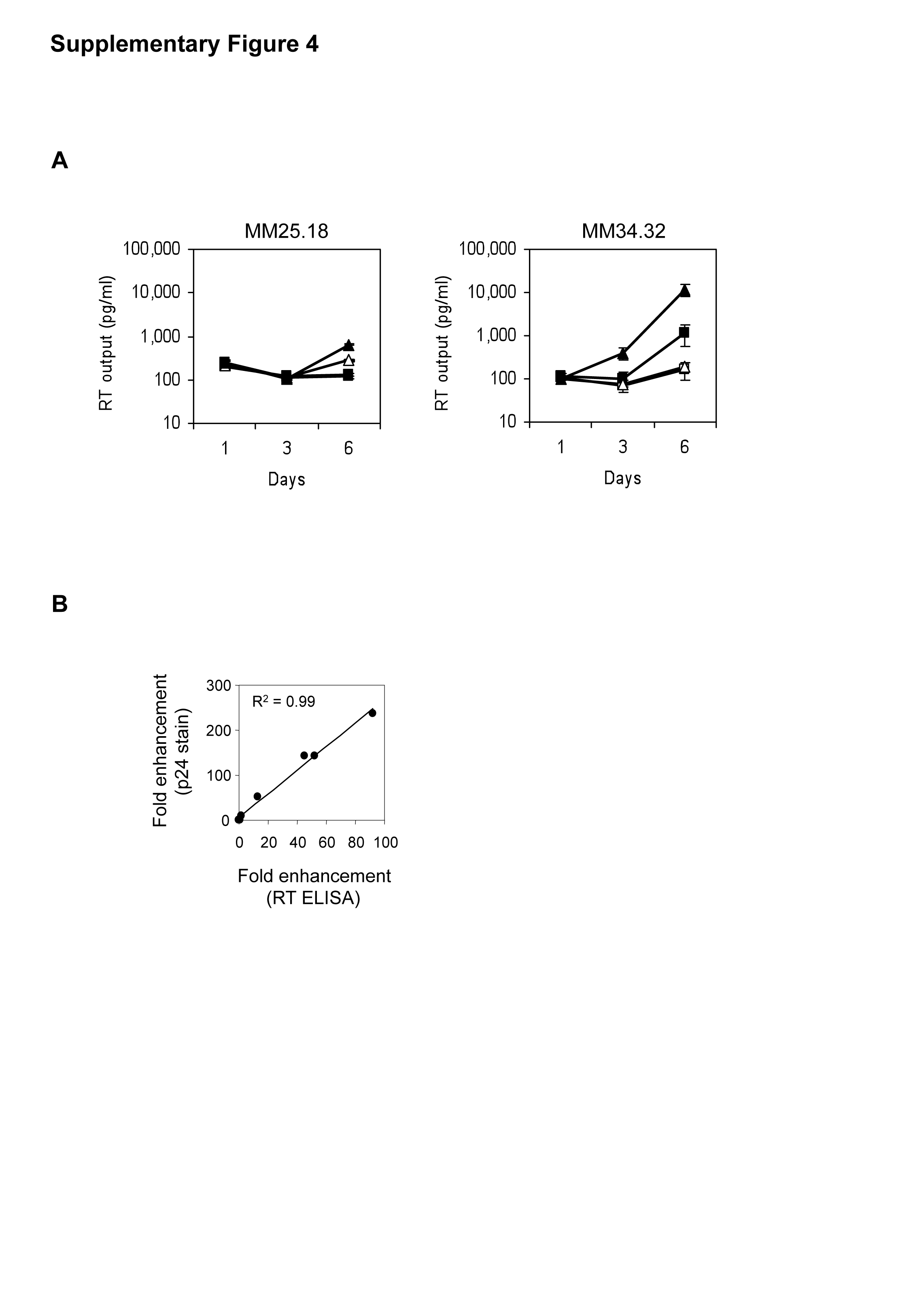

Supplement: Additional file 4 — Supplementary Figure 4. RT correlation and enhancement assay time course. In addition to % cells infected, fold enhancement was also measured using RT detected in the cell supernatant as a surrogate marker of virus production. (A) RT output was monitored in the cellular supernatant on days 1, 3 and 6 of enhancement assays for viruses showing high-level (MM34.32) and low-level (MM25.18) enhancement. Results are shown for MM25.18 virus with day 31 serum or NHS (left) and MM34.32 virus with day 25 serum or NHS (right). MM25.18 virus: when measured by RT output, day 31 serum enhanced infection 5.1-fold in the presence of C' (black triangles; 639 pg/ml RT) compared to NHS + C' (black squares; 127 pg/ml; 8.16-fold when measured by % cells infected, see Figure 2); whereas complement alone (NHS + C'; black squares; 127 pg/ml RT) enhanced infection 1-fold compared to NHS + HIC' (white squares; 125 pg/ml RT; 2.4-fold when measured by % cells infected, see Table 1). MM34.32 virus: when measured by RT output, day 25 serum enhanced infection 10-fold in the presence of C' (black triangles; 11,798 pg/ml RT) compared to NHS + C' (black squares; 1,183 pg/ml RT; 9-fold when measured by % cells infected, see Figure 2); whereas complement alone (NHS + C'; black squares; 1,183 pg/ml RT) enhanced infection 7.3-fold compared to NHS + HIC' (white squares; 162 pg/ml RT; 9.7-fold when measured by % cells infected, see Table 1). (B) Fold enhancement calculated from % cells infected by MM24.26 in the presence of 8 autologous sequential serum samples and complement (see Figure 2, MM24) was compared with fold enhancement attained by the same serum samples but calculated from RT output. [file 1742-4690-8-16-S4.TIFF]
